# The Selective Allure of Neuroscientific Explanations

**DOI:** 10.1371/journal.pone.0107529

**Published:** 2014-09-10

**Authors:** Nicholas Scurich, Adam Shniderman

**Affiliations:** 1 Department of Psychology & Social Behavior, Department of Criminology, Law & Society School of Law, University of California Irvine, Irvine, California, United States of America; 2 Department of Criminal Justice, Texas Christian University, Fort Worth, Texas, United States of America; University College London, United Kingdom

## Abstract

Some claim that recent advances in neuroscience will revolutionize the way we think about human nature and legal culpability. Empirical support for this proposition is mixed. Two highly-cited empirical studies found that irrelevant neuroscientific explanations and neuroimages were highly persuasive to laypersons. However, attempts to replicate these effects have largely been unsuccessful. Two separate experiments tested the hypothesis that neuroscience is susceptible to *motivated reasoning*, which refers to the tendency to selectively credit or discredit information in a manner that reinforces preexisting beliefs. Participants read a newspaper article about a cutting-edge neuroscience study. Consistent with the hypothesis, participants deemed the hypothetical study sound and the neuroscience persuasive when the outcome of the study was congruent with their prior beliefs, but gave the identical study and neuroscience negative evaluations when it frustrated their beliefs. Neuroscience, it appears, is subject to the same sort of cognitive dynamics as other types of scientific evidence. These findings qualify claims that neuroscience will play a qualitatively different role in the way in which it shapes people’s beliefs and informs issues of social policy.

## Introduction

Some claim that neuroscience will revolutionize the way we think about fundamental human nature. An editorial published in *The Economist* exemplifies this sentiment: “Genetics may yet threaten privacy, kill autonomy, make society homogeneous and gut the concept of human nature. But neuroscience could do all of these things first (p. 837).” [1] Such enthusiasm is not restricted to the mainstream media. Indeed, many academics have been enthralled by the transformative potential of neuroscience, and, as a result, neuroscience is now being used to study ‘old problems’ in a variety of disciplines, including philosophy [2], economics [3], marketing [4], management [5], and finance [6].

Legal scholarship is perhaps the most prominent area impacted by neuroscience. In a highly-influential article, neuroscholars Greene and Cohen [7] suggested that neuroscience will radically alter society’s moral intuitions about free will and responsibility, necessitating a change in the way criminal law deals with culpability and punishment. The result of this provocative article has been an influx of commentary about what implications neuroscience has for the law and the criminal justice system more broadly. In 2009 alone, there were more 200 law review articles that discussed neuroscience [8]. Neuroscience has also been credited by some with playing an influential role in a recent trilogy of juvenile justice cases heard before the United States Supreme Court [9].

A number of empirical studies have examined the influence of neuroscience on judgments and decisions. In the seminal article, *The Seductive Allure of Neuroscience Explanations* [10], researchers provided naïve adults, students in a neuroscience class, and neuroscience experts with sound or unsound explanations of psychological phenomenon that were either accompanied with a neuroscience explanation or not. While all participants were able to differentiate between the sound and unsound explanations when neuroscience was absent, non-experts found both explanations equally believable and satisfying when neuroscience was present. However, experts correctly detected that the neuroscience was irrelevant. Thus, the results appeared to support the contention that laypeople are bamboozled by neuroscience. Notably, since its publication, the article has been cited over four hundred times.

A second article, *Seeing is Believing: The Effect of Brain Images on Judgments of Scientific Reasoning* [11], extended this line of inquiry by providing participants with neuroscience data accompanied by images of the brain. In this study, university undergraduates read summaries of cognitive neuroscience research, which was accompanied by either a bar graph depicting the results, a brain image depicting areas of activation, or nothing. While the bar graph and control conditions did not differ, the addition of the brain image significantly increased judgments of scientific credibility. Across three separate studies, the authors noted, “that there is, indeed, something special about the brain images with respect to influencing judgments of scientific credibility (p. 350)”, and they speculated “that brain images are influential because they provide a physical basis for abstract cognitive phenomenon (p. 343).”

These two studies were the impetus for a cottage industry of research testing the effects of neuroscience explanations and images in a variety of contexts, most conspicuously the legal context. These studies have yielded mixed results that appear to depend on the specific purpose for which the neuroscience is proffered [12]–[17]. More pointedly, attempts to replicate and extend the original *Seductive Allure* and *Seeing is Believing* findings have been mixed or unsuccessful [18]–[20].

### The Present Study

Virtually all of the prior empirical research examining the impact of neuroscience has been atheoretical. In contrast, we hypothesize that neuroscience is subject to a cognitive dynamic known as *motivated reasoning* [21]. Motivated reasoning refers to the unconscious tendency to assimilate information in a manner biased towards reaching a particular outcome. This dynamic is more than simply making self-serving attributions; it unknowingly affects the way in which information is processed. Information that is congenial to a preferred outcome gets credited while information that does not facilitate the preferred outcome gets ignored or discredited. This process leads individuals to reach the desired outcome and it enables them to believe that their assessment is objective and rational [21], [22].

Empirical support for this phenomenon is voluminous [23]. In a classic study from the 1950s, students from two different Ivy League schools were asked to evaluate controversial calls made by an official during a football game; the students evaluated the legitimacy of the calls in a manner that was favorable to their institution, despite claims that their evaluations were neutral and impartial [24]. In another classic study, Lord, Ross, and Lepper [25] randomly assigned students to read about a fictional study that either did or did not find a deterrent effect of capital punishment. The content of the study was held constant, while the outcome of the study was manipulated. Students accepted the study at face value and deemed the methodology sound when the outcome of the study was consistent with their prior beliefs about the death penalty, yet they heavily scrutinized the study when it was inconsistent with their prior beliefs. Koehler [26] replicated this effect with scientists evaluating the quality of scientific issues, and the phenomenon has been replicated in numerous domains including medical testing [27], juror decision making [28], gambling [29], anthropogenic climate change [30], and even scientific peer review [31].

Based on this research, we predict that, rather than a universal seductive allure, neuroscience will have a ‘selective’ allure depending on how it corresponds to one’s predisposition about the issue for which it is proffered. Specifically, neuroscience will be appealing when it supports one’s predisposition but not convincing when it is incongruent with one’s predisposition. Such a finding would qualify claims that, in the eyes of the public, neuroscience will be able to definitively settle fundamental questions about human nature [32]. It would also replicate the phenomenon of motivated reasoning in a new domain.

## Study 1. Neuroscience and the Death Penalty

### Method

The University of California–Irvine Institutional Review Board (UCI IRB) approved the methodology described below (HS #2012–9060). Participants provided written consent by clicking on a button, which recorded the participant’s consent. The UCI IRB approved this procedure.

#### Participants

Participants were recruited through Amazon Mechanical Turk [33], which provides an online forum to access individuals interested in completing tasks for a nominal fee. Common tasks posted on the forum include surveys, questionnaires, and market research questions about products and websites. Our task required participants to be at least 18 years old and a citizen of the United States, and they were paid $1.00 for their participation.

A statistical power analysis assuming a medium effect size with a type-1 error rate of .05 and 80% power requires a sample size of 128. Because we were unsure how the sample would split with respect to their view on the death penalty, we collected responses from 170 participants. As described below, 19 participants were removed from the analyses and one participant withdrew halfway through the study. This yielded a final sample of 150 United States citizens of which 48% (n = 72) was female. Age of participants ranged from 18 to 62 with a median of 30 and interquartile range of 11. Forty-four percent (n = 67) of participants self-identified as a Democrat, 10% (n = 15) as a Republican, and 31% (n = 46) as an Independent, 8% (n = 8) as “other” and 9% (n = 14) as “none.”

### Procedure

The study began by asking participants a series of demographic questions and questions about social issues, such as the use of the death penalty for convicted murders, the legal propriety of abortion, and the vaccination of school-aged girls for the Human Papillomavirus, all of which were rated on a 5-point likert scale ranging from strongly oppose to strongly support. The order of the questions was randomized. Participants were then informed that they would be presented with a newspaper article and asked for their thoughts on the article. The newspaper article was formatted to emulate a traditional in-print article.

The newspaper article described the results of a fictitious experiment in which violent offenders were randomly assigned to one of two possible conditions. In the experimental condition, the offenders watched a video of an inmate being executed by lethal injection. In the control condition, the offenders watched a video of an inmate being held in solitary confinement. After watching the video both groups of offenders had their brain scanned using functional Magnetic Resonance Imaging (fMRI). The outcome of the fMRI scan was experimentally manipulated. Participants were either told that a) the fMRI detected significant differences in the amount of activation in the prefrontal cortex between the two conditions, or b) that the fMRI detected no differences in activation between the groups. The lead author of the hypothetical study–a researcher at a prestigious university–explained that the prefrontal cortex is related to impulsivity and aggressiveness. He then opined that the results of the study either support the notion that the death penalty is or is not an effective deterrent to crime. Thus, participants in the present experiment were randomly assigned to one of two possible conditions: neuroscience indicates the death penalty is a deterrent (hereinafter “is a deterrent”) or neuroscience does not indicate that it is a deterrent (hereinafter “not a deterrent”).

Following the newspaper article, participants were presented with two reading comprehension questions. The first asked about the subject of the article (i.e., the death penalty) and the second asked about the type of science that was described in the article (i.e., neuroscience). Participants who failed to correctly answer these questions (n = 19) were eliminated from the analyses reported herein [34]. Participants were then asked 10 questions about the validity of the study reported in the newspaper article (e.g., “How scientific was the study described in the article?” “How likely are the results reproducible in future studies?” “How persuasive was the neuroscience reported in the study?”) and about neuroscience in general (“Is neuroscience is limited like other forms of social science?” “Is neuroscience science relevant to social policy?”). All ratings were made on a 7-point likert scale from strongly disagree to strongly agree and some were reverse scored. The order of the questions was randomized.

## Results

A reliability analysis conducted on the 10 response questions to the newspaper article yielded a Cronbach’s alpha = .891. The 10 items were collapsed into a composite score, referred to as “neuroscience quality,” and the composite score was then transformed into a z-score. As measured at the pre-test stage of the study, 37% (n = 56) of participants oppose the death penalty, 17% (n = 25) were neutral about the death penalty, and 46% (n = 69) support the death penalty. Neutral participants were omitted in the following analyses.

**Figure 1:**
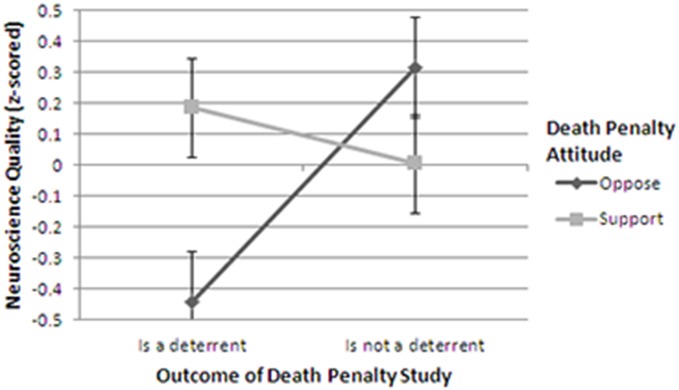
Perceptions of the quality of neuroscience as a function of the outcome of the study and participants’ a priori attitudes towards the death penalty. Note that error bars are +/−1 S.E.

A 2-way ANOVA with ‘result of study’ (is a deterrent or is not a deterrent) and pre-test ‘death penalty attitude’ (oppose or support) as the independent variables and neuroscience quality as the dependent variable detected a significant interaction F(1, 125) = 8.02, p = .005, η_p_^2^ = .062. The main effect for death penalty attitude was not significant (F(1, 125)<1) nor was the main effect for result of study (F(1, 125) = 3.0, p = .086, η_p_^2^ = .024). These findings are displayed graphically in Figure 1.

As is apparent in figure 1, participants who oppose the death penalty (the black line) gave much higher ratings to the neuroscience when the study purported to find that the death penalty is not a deterrent to crime than when the study purported to find a deterrent effect. The opposite pattern emerged for participants who support the use of the death penalty (the grey line). They rated the quality of the neuroscience high when the purported to find a deterrent effect and low when it did not find a deterrent effect.

Note that conducting a median split to parse participants based on their pre-test attitudes towards the death penalty – as opposed to omitting the neutral participants – does not materially affect the results: A 2-way ANOVA detected a significant interaction (F(1, 150) = 7.35, p = .008, η_p_^2^ = .048). The main effects for death penalty attitude and result of study were not significant (F(1, 150)<1 and F(1, 150) = 2.48, p = .12, η_p_^2^ = .017, respectively).

## Discussion

The findings from study 1 are consistent with the selective allure hypothesis. Participants credited the neuroscience and gave it favorable ratings when it supported their attitude towards the death penalty, and they denigrated the science when it conflicted with their attitude towards the death penalty. These findings suggest that neuroscience is not unlike other forms of science in the way in which it is consumed by the lay public. In the same way that people selectively scrutinize social science and physical science, people also selectively scrutinize neuroscience. Indeed, the study replicates the findings of Lord, Ross, & Lepper [25] but with the use of neuroscience instead of conventional social science. Thus, claims that neuroscience is categorically different than previous forms of research, perhaps because of its unique ability to pinpoint specific regions of the brain that are responsible for behavior [35], are not supported by the current study.

## Study 2. Neuroscience and Abortion

The previous study looked at the perception of neuroscience as it pertains to one particular social issue. It is possible that the death penalty is unlike myriad other social issues. Thus, a second study was conducted to test whether the phenomenon would replicate across different social issues with a different sample of participants. The methods of the second study were nearly identical except for the content of the newspaper articles.

### Method

#### Participants

Participants were again recruited through Amazon Mechanical Turk. They were required to be at least 18 years old and a citizen of the United States, and were paid $1.00 for their participation. A software setting precluded study 1 participants from engaging in this study. As described below, 14 participants were removed from the analyses for failing one of the comprehension check questions, which yielded a sample of 149 participants, of which 47% (n = 70) was female. Age of participants ranged from 19 to 68 with a median of 32 (IQR = 18). Forty-five percent (n = 68) of participants self-identified as Democrats, 11% (n = 16) as Republicans, and 32% (n = 47) as Independents, 3% (n = 5) as “other,” 8% (n = 12) as “none,” and one person identified as a Tea Party member.

### Procedure

Consistent with the previous study, participants were initially asked a series of demographic questions and questions about social issues, all of which were presented in random order. Participants were then informed that they would be presented with a newspaper article and asked for their thoughts on the article.

The newspaper article began by explaining that, although abortion is a divisive political issue, most agree that abortion should be legally prohibited if the fetus experiences pain during the procedure. The article then described a study in which fetuses experienced an ultrasonic sound while being scanned by an fMRI. This sound is known to arouse slight discomfort and pain in babies less than one year of age. The lead researcher explained that activation in the parietal lobe indicates the sensation of pain, and thus fMRI can answer the question of whether fetuses experience pain by whether activation is detected.

The outcome of the study was experimentally manipulated. In one condition (referred to as “pain”), the researchers found that second and third trimester fetuses displayed activation in the parietal lobe, and are thus capable of feeling pain. A fictional figure in the pro-life movement then opined that the study suggests that second trimester abortions should not be legally permitted because the fetus feels pain. In the other condition (“no pain”), the fMRI scan revealed no activation in the parietal lobe of second trimester fetuses. A fictional figure in the pro-choice movement then opined that the study suggests that second trimester abortions should be legally permitted because the fetus does not feel pain.

Following the newspaper article, participants were presented with two reading comprehension questions. The first asked about the subject of the article (i.e., abortion) and the second asked about the type of science that was described in the article (i.e., neuroscience). Participants who failed to correctly answer these questions (n = 14) were eliminated from the analyses reported herein [34]. Participants were then asked 10 questions in random order about the validity of the study reported in the newspaper article and about neuroscience in general. All ratings were made on a 7-point likert scale from strongly disagree to strongly agree and some were reverse scored.

## Results

A reliability analysis conducted on the 10 response questions yielded a Cronbach’s alpha = .874. Removing any item would not significantly increase alpha. The 10 items were collapsed into a composite score, hereinafter referred to as “neuroscience quality,” which was transformed into a z-score. As measured at the pre-test stage of the study, 27.5% (n = 41) of participants oppose abortion, 14% (n = 21) were neutral, and 58.5% (n = 87) support abortion. Neutral participants were removed from the following analyses.

**Figure 2:**
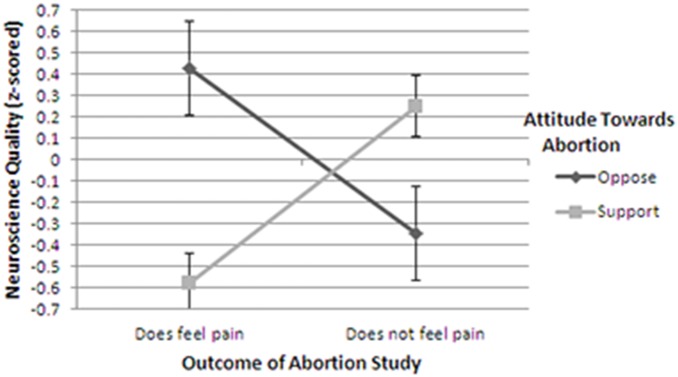
Perceptions of the quality of neuroscience as a function of the outcome of the study and participants’ a priori attitudes towards abortion. Note that error bars are +/−1 S.E.

A 2-way ANOVA with ‘outcome of study’ (fetus feels pain or fetus does not feel pain) and pre-test ‘abortion attitude’ (oppose or support) as the independent variables and neuroscience quality as the dependent variable only detected a significant interaction F(1, 128) = 17.73, p<.001, η_p_^2^ = .125. The main effect for death penalty attitude was not significant (F(1, 128)<1) nor was the main effect for result of study (F(1, 128) = 1.19, p = .28). These findings are displayed graphically in Figure 2.

As is apparent, the perceived quality of the neuroscience in the study depended on both the outcome of the study and how that outcome related to participants’ attitudes towards abortion. When the study indicated that fetuses experience pain, and therefore abortion should be proscribed at the second trimester, participants who oppose abortion (the dark bar) rated the quality of the neuroscience quite high, while participants who support abortion (the light bar) rated it relatively low. The converse pattern of results appeared when the outcome of the study indicated that second trimester fetuses do not experience pain. In that case, participants who support abortion gave the neuroscience high quality-ratings while those who oppose abortion gave it relatively low quality-ratings.

Note that conducting a median split to parse participants based on their pre-test attitudes towards abortion –as opposed to omitting the neutral participants–does not materially affect the results: A 2-way ANOVA detected a significant interaction (F(1, 150) = 18.43, p<.001, η_p_^2^ = .113). The main effects for abortion attitude and result of study were not significant (both Fs(1, 149)<1).

## General Discussion

A 2008 study conducted by the American Psychological Association on public perceptions of psychology found that large percentages of the public are skeptical about psychology’s scientific status. For instance, only 30 percent of respondents agreed with the statement, “psychology attempts to understand the way people behave through scientific research (p. 3).” [36] Neuroscience has been proposed as the scientific, objective solution to the study of human behavior [32]. Two independent experiments unequivocally supported the selective allure hypothesis. Whether neuroscience can provide the sort of objective truth that some commentators advert to, neuroscience is apparently not treated as objective by laypersons. Consistent with copious research from other domains, neuroscience is more likely to be accepted and credited when it confirms prior beliefs. Thus, while neuroscience might be viewed as more scientific in the abstract, it is unlikely to play a qualitatively different role in the way in which it shapes people’s beliefs and informs issues of social policy.

As with any experimental endeavor, the findings should be qualified and the limitations should be acknowledged. The representativeness of the Amazon Turk Worker pool is not well-known [37] and there is the possibility that the most ardent participants self-selected to participate in experiments involving social and political issues. While potentially valid, this would suggest a reduction in the observed effect sizes rather than a change in their direction. Replication efforts with different samples and stimuli are clearly necessary. Further research is also necessary to examine the boundary conditions of this effect, especially when the issue to which neuroscience is relevant is less politically polarizing. The observed effect would likely be attenuated for non-moral, or less ideologically and politically charged issues [38] but still likely to occur to some degree [39]–[40].

One should bear in mind that the substance of each of the hypothetical studies was identical in the two experimental conditions; the only difference was the outcome of the study. While a single study is unlikely to impel people to completely revise their attitude about a controversial social issue, selectively crediting or discrediting scientific studies based on whether they support previous beliefs virtually ensures that such beliefs will remain largely unchanged. There is some debate as to whether it is logically appropriate for the evaluation of scientific study (or information, more generally) to depend on the outcome it furnishes [26]. We take no position on this normative issue. However, we do note that this reasoning can lead to an echo chamber that contributes to and galvanizes political polarization, and that such reasoning can be especially pernicious if prior beliefs are objectively wrong. Further research might examine potential ways to ameliorate the selective allure effect. Lord, Ross, and Preston [41] found that providing instruction to consider both sides of the argument significantly reduced the tendency to selectively credit information. The possibility of this and other remedies is promising but ultimately an empirical question.

### Final Thoughts

Over 35 years ago, Lord, Ross, and Lepper [25] poignantly observed that, “If our study demonstrates anything, it surely demonstrates that social scientists cannot expect rationality, enlightenment, and consensus about policy to emerge from their attempts to furnish ‘objective’ data about burning social issues (p. 2108).” The present findings suggest that this observation applies to neuroscience too. Although hope has been expressed to the contrary, neuroscience –like conventional social science–does not appear to be the panacea for resolving social policy issues that are based on assumptions about human behavior.
